# Supplementary material for: Kosterlitz-Thouless melting of magnetic order in the triangular quantum Ising material TmMgGaO4
Source: Nat Commun. 2020 Feb 28;11:1111. doi: 10.1038/s41467-020-14907-8 (PMC7048727; doi:10.1038/s41467-020-14907-8)
Supplement: Supplementary file 1 — Supplementary Information [file 41467_2020_14907_MOESM1_ESM.pdf]

Supplementary Information for

Kosterlitz-Thouless Melting of Magnetic Order in the Triangular Quantum Ising Material $TmMgGaO_4$

Li *et al*.

Supplementary Note 1: The electron density distributions and magnetic interactions in TMGO.

Density-functional theory (DFT) calculations of TMGO can be performed via the Vienna *ab initio* simulation package, with the projector augmented wave method [1, 2]. In the DFT calculations, we take the TMGO lattice parameters $a = b = 3.4260$ Å and $c = 25.1690$ Å as determined from experiments.

When Coulomb interaction is switched off ($U = 0$), the band structure of TMGO indicates a metallic state [Supplementary Fig. 1(a)], while a finite U (= 3 eV) opens up a Mott gap as shown in Supplementary Fig. 1(b). The partial electron density relevant for magnetic exchange interactions include the contributions from 4f electrons of Tm^{3+} and 2p electrons of O^{2-}, as seen in the density of states in Supplementary Figs. 1(a,b). The Tm-O-Tm superexchange paths are visualized as electron clouds overlap in Supplementary Fig. 1(c), and the Tm^{3+} ions form a triangular lattice [lower plot in Supplementary Fig. 1(c)]. The two dimensionality of the material TMGO is manifested, as the electron density at the interlayer regime is negligibly small compared to that in the Tm-O-Tm path.

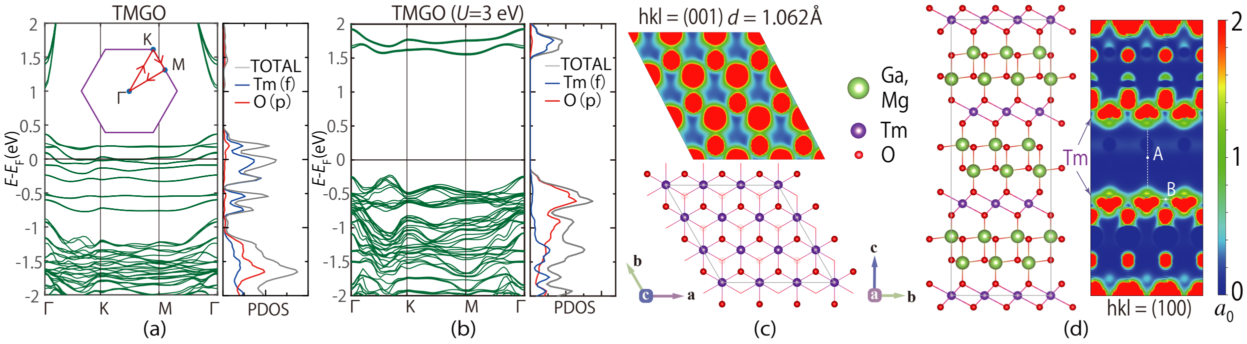

Supplementary Figure 1. **The crystal structure and partial electron density distributions.** The energy band results are plotted with (a) $U = 0$ and (b) $U = 3$ eV, where the corresponding densities of states (total, 4f, and 2p) are also shown. (c) depicts the layered lattice structure and corresponding electron density of TMGO [in the unit $a_0 = 10^{-6}$ e·Bohr^{-3}, and note the density plot (above) corresponds precisely to the regime enclosed by the black diamond-shape box in the lattice structure (below)]. The partial electron density ρ_e with energies between 1.5 and 2 eV (in the $U = 3$ eV calculations) includes mainly 4f and 2p electron contributions. (d) provides a side view of partial electron density ρ_e, where we find ρ_e at point A (between two layers) and B [a typical point in the superexchange path within the (001) plane] are different by 2-3 orders of magnitude, manifesting the two dimensionality of magnetic couplings in TMGO.

Supplementary Note 2: Spin ordering and phase diagram of the J_1-J_2 TLI.

As mentioned in the main text, by increasing the ratio of J_2/J_1 we can drive the system from the clock order phase to a stripe order phase. To verify this, firstly we calculate the static magnetic structure

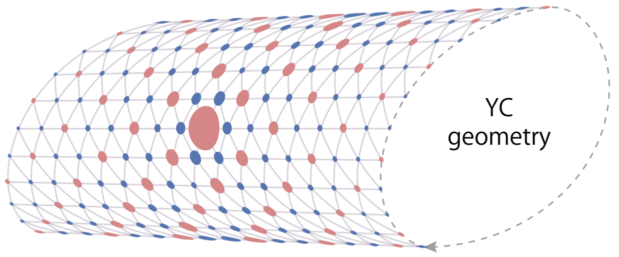

Supplementary Figure 2. **Real-space correlation $\langle S^z_0 \cdot S^z_{\rm r}\rangle$ and a three-sublattice order.** Each solid circle represents the real-space $\langle S^z_0 \cdot S^z_{\rm r}\rangle$ correlation related to the central site 0, the size of circle denotes the magnitude of correlation, and the red(blue) color for the positive(negative) sign. The clock order pattern with an enlarged unit cell can be clearly seen. The XTRG calculation is performed with parameters $J_1 = 0.99$ meV, $J_2 = 0.05J_1$, and $\Delta = 0.54J_1$ at $T \simeq 0.57$ K, on the YC geometry also specified in the plot.

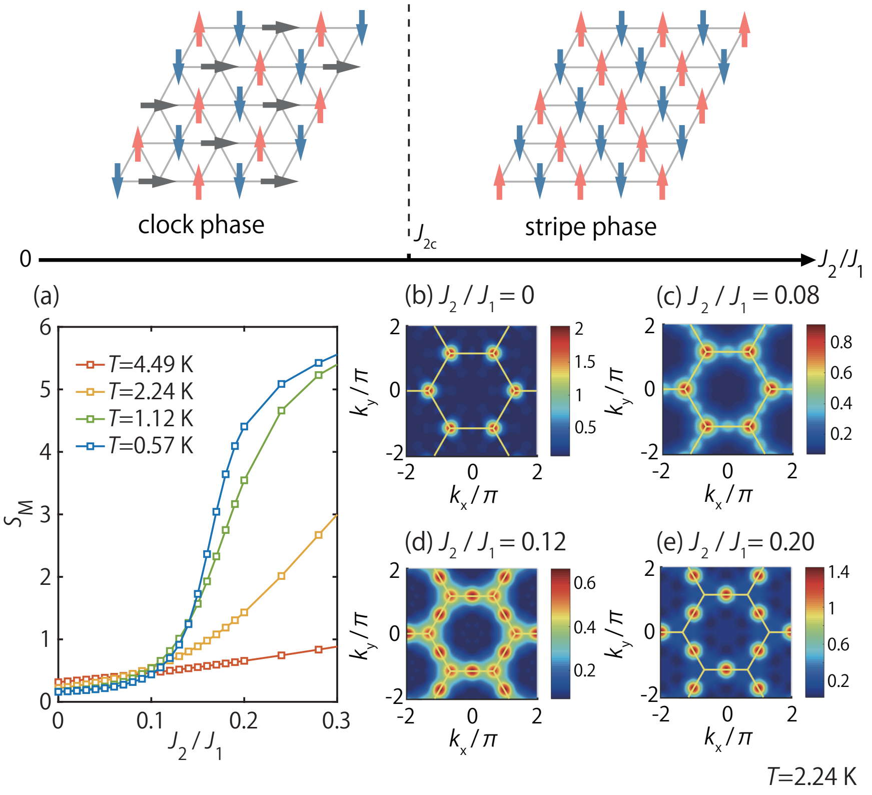

Supplementary Figure 3. **Schematic phase diagram of the J_1-J_2 TLI and static structure factors.** As J_2 increases, there exists a quantum phase transition between the clock and stripe phases, taking place at $J_{2c}/J_1 \sim 0.1$ (for $\Delta/J_1 = 0.54$). In (a) we collect the M-point intensity and plot it vs. J_2/J_1 at four different temperatures, from which we see clearly that $S_{\rm M}$ in small J_2 regime is continuously connected to that in the relatively large J_2 regime, i.e., the stripy phase. The contour plots of $S(q)$ are shown in panels (b-e), with various J_2/J_1 values (0 to 0.2). In the calculations, we fix the parameter as $\Delta/J_1 = 0.54$, $J_1 = 0.99$ meV, and compute $S(\mathbf{q})$ at T = 2.24 K for (b-e).

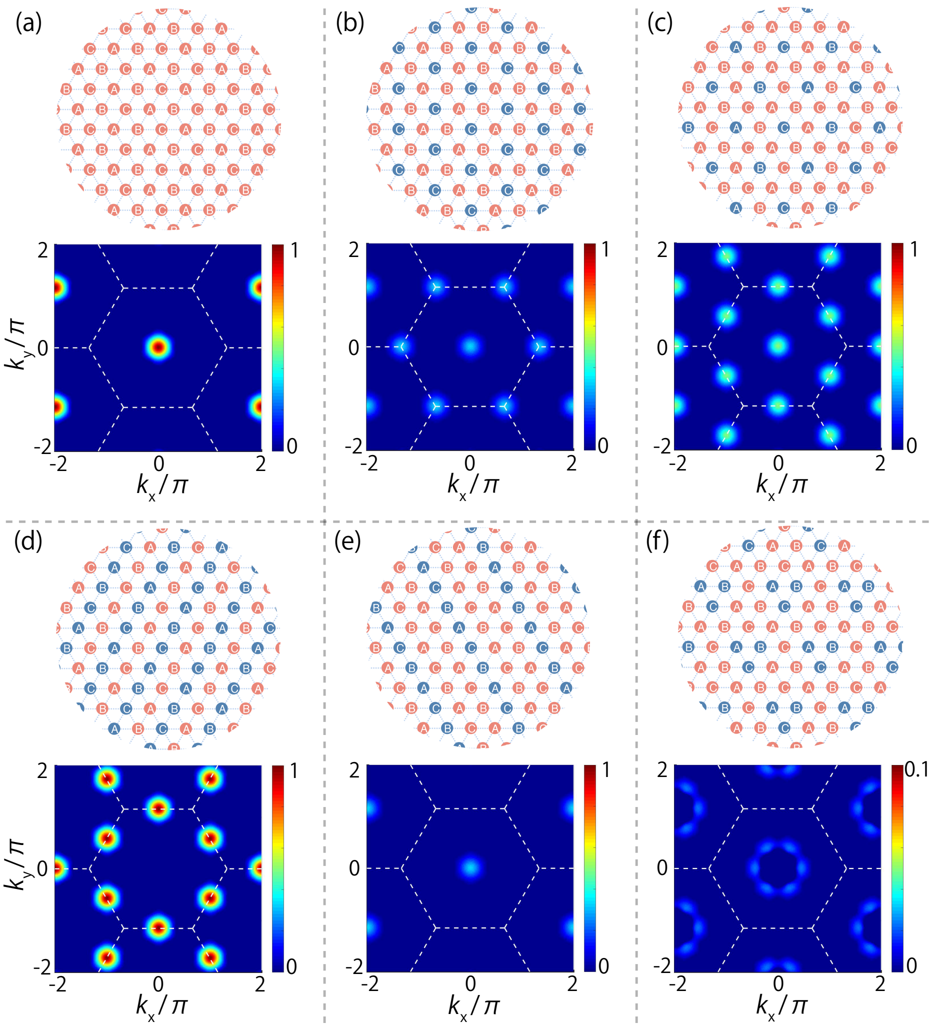

Supplementary Figure 4. Classical orders of the $J_1 - J_2$ TLI model and their static structure factor $S(\mathbf{q})$. Panels (a-f) plot six different magnetic orders (all possible classical spin configurations from Supplementary Ref. [3]), along with the corresponding spin structures in reciprocal space. Note the $S(\mathbf{q})$ data are computed in a 12 × 12 cluster, symmetrized, and normalized by the number of sites.

factor $S(\mathbf{q})$ from $\langle S_0^z S_r^z \rangle$ correlations, and show the results in Supplementary Fig. 2. From the real-space correlations on YC6 geometry, a three-sublattice order can be clearly identified, which translates into the $S(\mathbf{q})$ peak at the K point [see, e.g., Fig. 5(b) in the main text, or Supplementary Fig. 3], signifying the presence of clock order.

However, as J_2/J_1 increases to 0.2, $S(\mathbf{q})$ changes its peak to the M point [Supplementary Fig. 3(e)], consistent with a two-sublattice stripe-order pattern. Therefore, there must be a quantum phase transition between the clock and stripe phases, probably of first order. As the stripe order (with structure factor peak at M point) is in close proximity to the clock phase (a small J_2 drives the phase transition), we relate the finite energy M-roton excitations in the dynamic spin spectra with instability towards the stripe order.

Supplementary Note 3: Classical spin orders and their static structure factors.

The classical spin orders of $J_1 - J_2$ TLI has been throughly investigated in Supplementary Ref. [3]. Here we replot the possible classical spin configurations in Supplementary Fig. 4, along with their computed static structure factor $S(\mathbf{q})$. One can see clearly that only orders in Supplementary Figs. 4(c,d) have peaks at M point. Supplementary Fig. 4(d) corresponds to the stripy order, in agreement with our simulated data (with large J_2), while the one in (c) has a Γ peak that is absent in our results. Therefore, the only classical Ising configuration that corresponds to our structure factor data is Supplementary Fig. 4(d), i.e., stripy order.

The other way around, in the context of our TLI model, Bragg peak at M $= (1/2, 1/2)$ corresponds to a π phase shift, i.e., antiferromagnetic correlation along primitive vectors $\mathbf{a}$ and $\mathbf{b}$ (see primitive vector in Fig. 1 of the main text), while the correlation is ferromagnetically along $\mathbf{a} + \mathbf{b}$ and $\mathbf{a} - \mathbf{b}$. This clearly corresponds to a magnetic stripy phase, in the context of TLI model.

Furthermore, we can also argue that large $J_2 > 0$ favors a stripe order: In the presence of J_2, the stripe Ising configuration [Supplementary Fig. 4(d)] leads to an energy estimate of $-J_1 - J_2$, while the up-up-down (UUD) order [A,B sublattice spin up and C sublattice down, see Supplementary Fig. 4(b)] $-J_1 + 3J_2$, and the order in Supplementary Fig. 4(c) 0 energy. Therefore, it is clear that the stripy configuration is energetically more favorable in large J_2 limit. Note that in a recent study of triangular lattice magnet $AgNiO_2$, the existence of stripe order was observed, which is ascribed to the relatively large J_2 in the compound [4].

Supplementary Note 4: TLI parameter fittings.

The parameter fitting workflow is as follows: we scan the parameters (J_1, J_2, Δ) to fit the specific heat $C_\mathrm{m}(T)$, magnetic entropy $S_\mathrm{m}(T)$, as well as the susceptibility $\chi(T)$ (at a small magnetic field h=1 kOe), and find the optimal parameters. Given that, we compute the magnetization curves (at different T) and magnetic entropy S_m at finite fields h, and compare directly to experimental data [5, 6], so as to ensure that the parameter set is adequate and precise to model the material.

To be concrete, we show in Supplementary Fig. 5 part of simulation data in our scanning. In Supplementary Fig. 5(a), we start with $J_2 = 0$ and scan various Δ values. It is found that C_m curves are sensitive, in terms of the peak height as well as the overall shape, to different Δ values. By tuning Δ (while keeping $J_2 = 0$), we find $\Delta/J_1 = 0.4$ and $J_1 = 1.1$ meV can produce results in agreement with experimental C_m curves. However, with this set of parameters (as well as other Δ values) we clearly miss the experimental susceptibility line, as plotted in Supplementary Fig. 5(b). It therefore suggests that a finite J_2 should be involved in the fittings.

After a thorough scanning in the parameter space, we find $\Delta/J_1 = 0.54$ with $J_2/J_1 = 0.05$ can well

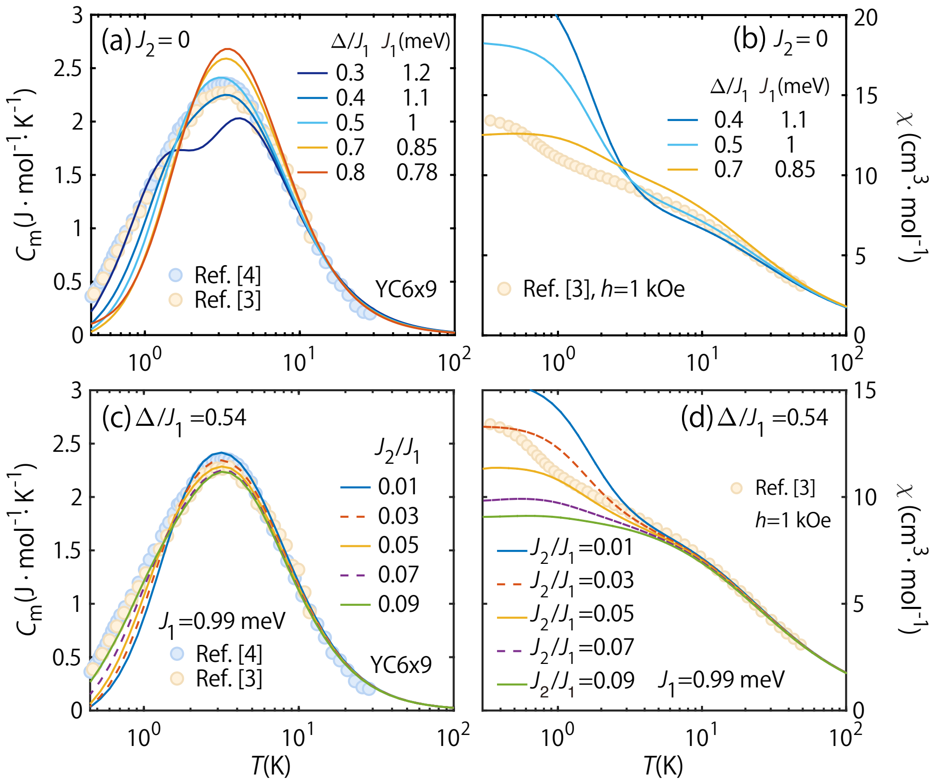

Supplementary Figure 5. **Parameter fittings.** (a) The specific heat $C_{\rm m}(T)$ curves with various Δ/J_1 and fixed J_2 = 0, obtained by XTRG simulations on the YC6×9 lattice. In panel (b) we show the susceptibility $\chi(T)$ with various Δ/J_1 = 0.4, 0.5, 0.7 (at h=1 kOe), which all clearly failed to fit the experimental data well. (c) shows the $C_{\rm m}(T)$ results with J_2/J_1 ranging from 0.01 to 0.09 and a fixed (optimal) Δ/J_1=0.54, and (d) depicts the susceptibility data correspondingly. $g_{\|} = 13.212$ is fixed throughout the fittings (see Supplementary Note 3).

reproduce both the specific heat curve in Supplementary Fig. 5(c) and the magnetic susceptibility data in (d). To show how sensitive the fittings are with respect to J_2, we also provide in Supplementary Figs. 5(c,d) the simulated data with $J_2 = 0.01$ to 0.09, from which we see that J_2 considerably influences both $C_m(T)$ and $\chi(T)$ curves. Regarding error bar of the fitted parameter J_2, the most probable regime where J_2/J_1 resides is between 0.03 and 0.05 (while 0.05 is still more preferable).

With this parameter set $J_1 = 0.99$ meV, $\Delta/J_1 = 0.54$, and $J_2/J_1 = 0.05$ (as well as $g_{\|} = 13.212$), we have computed the magnetization curves at two different temperatures and entropy $S_{\rm m}$ at finite magnetic fields. We compare them to experimental data in Fig. 2 of the main text and observe excellent agreement. Remind that there we push the calculations to YC9 lattice with width $W = 9$, and the fittings are equally good, suggesting the robustness of fittings vs. system sizes. Beyond equilibrium properties, we have also computed dynamical properties $\omega(k)$ with same parameter set on a 36×36 lattice, which also show excellent agreement with experimental data (e.g., the overall dispersion line and gap values). These direct

comparisons lead us unambiguously to the conclusion that the above parameters of TLI can describe the material TMGO precisely.

Supplementary Note 5: The Curie-Weiss Fitting of high-T susceptibility.

As a complementary of the thermodynamic fittings in the main text, in Supplementary Fig. 6 we compare the experimental and simulated high-temperature magnetic susceptibility. It is found that the XTRG data lie on top of two experimental curves, with fitted $\Theta_{\rm W} \simeq 19.3$ K, in very good agreement with the estimates in experimental works (e.g., 18.9 - 19.1 K as indicated in the plot). Besides, the fitted constant $C \simeq 210.1$ cm^{3}K/mol leads to an estimate of the effective $g_{\parallel} = \frac{1}{\sqrt{S(S+1)}\mu_{\rm B}} \frac{\sqrt{3k_{\rm B}C}}{\sqrt{N_{\rm A}\mu_0}} \simeq 13.212$, where $S = 1/2$ represents the effective spin-1/2, which is in excellent agreement with the various thermodynamic fittings till low temperatures in the main text and thus constitutes a self-consistency check. Remind this $g_{\parallel}$ value obtained is in reasonably good agreement with the ideal Landé factor $g_J = 7/6$ (ideally $J_{\parallel} = 2Jg_{\rm J}$).

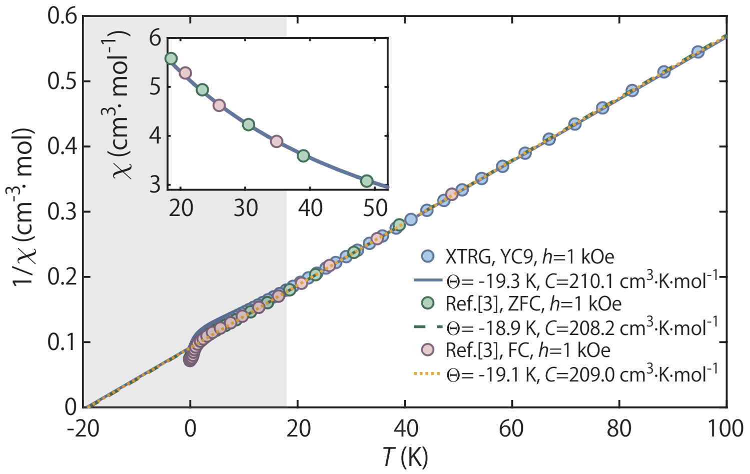

Supplementary Figure 6. **The Curie-Weiss behavior at high temperature.** Here we show the high-T susceptibility data and analyse the Curie-Weiss behavior $\chi = \frac{C}{T-\Theta}$, where the inverse susceptibility χ^{-1} vs. T is plotted in the main panel and the χ vs. T curve is plotted in the inset (with Curie-Weiss fitting lines also shown). The XTRG susceptibility χ is computed with the model parameters $J_1 = 0.99$ meV, $J_2 = 0.05J_1$, $\Delta = 0.54J_1$, and $h = 1$ kOe, on a YC9 $\times$ 12 lattice. The Curie-Weiss fittings are done within the temperature range $T \in$ [6, 48] K (experimental curves, FC and ZFC), and $T \in$ [18, 100] K (XTRG data), i.e., high temperature data on the right of the grey shaded regime.

To conclude, through large-scale simulations of both equilibrium and dynamical properties, we pinpoint the model parameters of TMGO as $J_1 = 0.99$ meV, $\Delta/J_1 = 0.54$, $J_2/J_1 = 0.05$, and $g_{\parallel} = 13.212$, which can be used to fit virtually all available experimental data, including the magnetic specific heat, entropy, susceptibility (both high- and low-temperature parts), and dynamical spin spectrum, etc.

Supplementary Note 6: Specific heat curves under external fields.

As noted in Fig. 2(d) earlier in the main text, the magnetization curves $M(h)$ show quasi-plateau structures at $M/M_{\rm sat} \simeq 1/3$, where $M_{\rm sat}$ is the saturation magnetization along the z direction. In Supplementary Ref. [5], the specific heat curves have also been measured under various external magnetic fields. In Supplementary Fig. 7(a), we redrawn the experimental data, and compare them, side by side, to the simulated $C_{\rm m}(h,T)$ curves. It can be seen that in both Supplementary Figs. 7(a) and (b), a low-T shoulder gradually appears under small fields, e.g., at $h = 5$ kOe, and then prominent peaks show up at around $h = 10$-20 kOe. By further increasing the magnetic fields, the peak height gets declined and at the same time its position moves towards higher temperatures for $h \geq 30$ kOe.

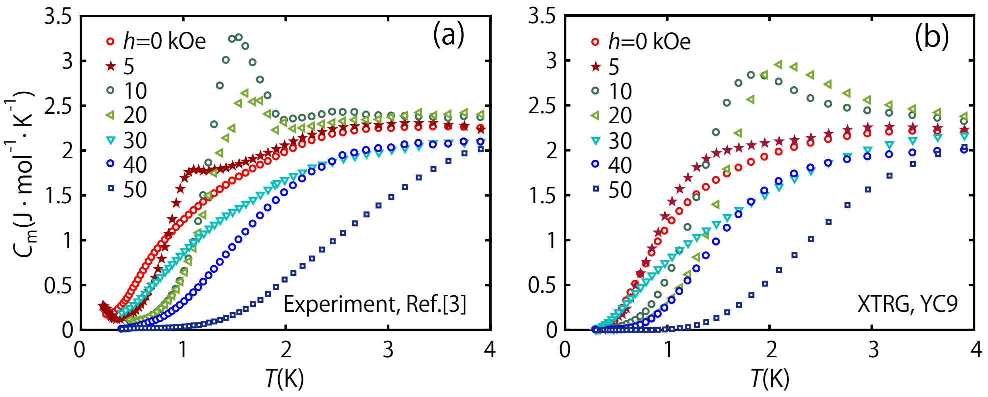

Supplementary Figure 7. **Specific heat curves under magnetic fields.** (a) shows the experimental specific heat data under various magnetic fields, taken from Supplementary Ref. [5], and (b) is the corresponding XTRG results. The comparisons are in quite remarkable consistency, suggesting the correctness of the parameter sets and the accuracy of XTRG calculations.

It is remarkable that these highly nontrivial and non-monotonic behaviors of $C_{\rm m}(h,T)$ curves can also be understood within the TLI model. As shown in Fig. 2(d) of the main text, there exists a quasi-plateau structure at about $1/3\, M_{\rm sat}$ in the curve, which suggests that the system undergoes a transition from the clock order to an UUD spin state upon increasing fields to $h = 10$-20 kOe. The forming of the UUD structure releases entropy and gives rise to the prominent low-T peak in $C_{\rm m}$, despite that the peaks in $C_{\rm m}$ between 1 and 2 K are less pronounced in the simulated results than experiments. As the field strength further increases, the UUD order becomes weaker and accordingly the $C_{\rm m}$ peak moves back to lower T side with a decreasing height. Eventually, for $h \geq 30$ kOe, the system gradually polarizes into a ferromagnetic spin configuration, and the hump in $C_{\rm m}$ moves towards higher T as h enhances.

Supplementary Note 7: Saddle point in the triangular tight-binding model.

When restricted in a subspace of configurations with only one pair of spins flipped (while others remain in the classical stripy order, in the small Δ limit), we consider a "tight-binding" model

$$H = \epsilon_0 + \sum_i \sum_\delta t(c_i^\dagger c_{i+\delta} + h.c.) \tag{1}$$

on the triangular lattice, where i labels the lattice site, and $|i\rangle$ labels a state with a spin flipped at site i. $\delta = \mathbf{a}, \mathbf{b}, \mathbf{a} - \mathbf{b}$ denotes nearest neighboring sites ($\mathbf{a}, \mathbf{b}$ are primitive vectors shown in Fig. 1 of the main text). Remember that a second-order process related to S^x terms in the Hamiltonian can actually tunnel between $|i\rangle$ and $|j\rangle$, so $t \sim \Delta^2$ (in the small Δ limit), given i and j constitute a pair of nearest neighboring sites.

For the sake of simplicity, we set $\epsilon_0 = 0, t = 1$, and take Fourier transformation of Eq. (1). The resulting dispersion $\epsilon(k) = 2[\cos(k \cdot \mathbf{a}) + \cos(k \cdot \mathbf{b}) + \cos(k \cdot (\mathbf{a} - \mathbf{b}))]$ is plotted in Supplementary Fig. 8(a). By cutting along the Γ-M-Γ path, we observe a quadratic low-energy dispersion near the minimum as shown in Supplementary Fig. 8(b). On the other hand, as shown in Supplementary Fig. 8(c) the M point constitutes a maximal along K-M-K path. Therefore, it is evident that the M point indeed constitutes a saddle point in the dispersion.

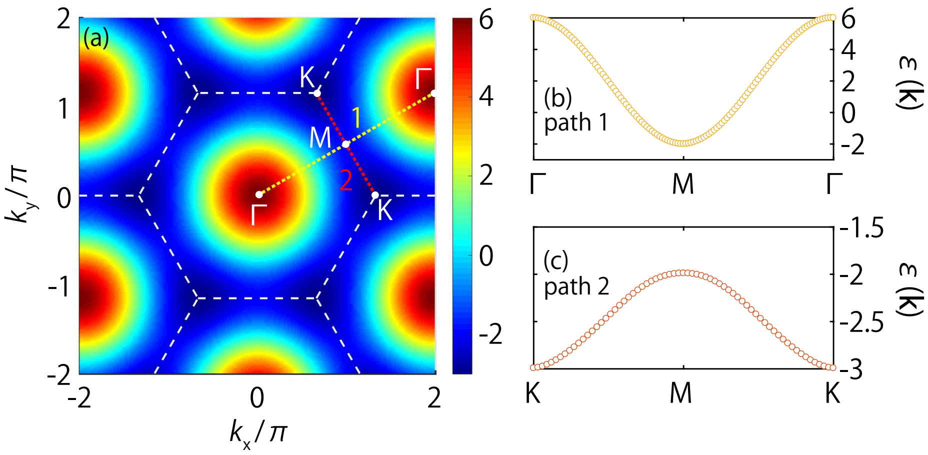

Supplementary Figure 8. **"Tight-binding" dispersion on triangular lattice and the saddle point at M.** (a) shows the contour plot of dispersion $\epsilon(\mathbf{k})$, and (b,c) show the cuts along paths 1 and 2, respectively.

Supplementary Note 8: A brief introduction of path integral QMC for TLI.

The Hamiltonian of quantum TLI is $\mathcal{H} = J_1 \sum_{\langle i,j\rangle} S_i^z S_j^z + J_2 \sum_{\langle\langle i,j\rangle\rangle} S_i^z S_j^z - h \sum_i S_i^x$, where the external magnetic field part is omitted for clearance of narrative. QMC evaluates the partition function $Z = \mathrm{Tr}\left[e^{-\beta\mathcal{H}}\right]$, where discretized imaginary time is used and $\beta = L_\tau \Delta\tau$ ($\Delta\tau = 0.05$). It maps the

problem onto a (2+1)D classical system as follows,

$$\begin{aligned} Z &= \text{Tr}\left[e^{-\beta\mathcal{H}}\right] \\ &= \text{Tr}\left[e^{-\Delta\tau\mathcal{H}}e^{-\Delta\tau\mathcal{H}}\cdots e^{-\Delta\tau\mathcal{H}}e^{-\Delta\tau\mathcal{H}}\right]. \end{aligned} \tag{2}$$

By inserting a complete set of S_{i}^z eigenstates between each pair of exponentials, i.e.,

$$\begin{aligned} \mathbf{1} &= \prod_{i=1}^{N}\left[\sum_{S_i^z=\pm 1}|S_i^z\rangle\langle S_i^z|\right] \\ &\equiv \sum_{\{S_i^z\}}|S^z\rangle\langle S^z|, \end{aligned} \tag{3}$$

we can then rewrite the partition function as,

$$\begin{aligned} Z = \sum_{\{S_{i,l}=\pm 1\}} & \langle S_1^z|e^{-\Delta\tau\mathcal{H}}|S_L^z\rangle\langle S_L^z|e^{-\Delta\tau\mathcal{H}}|S_{L-1}^z\rangle\langle S_{L-1}^z|e^{-\Delta\tau\mathcal{H}}|S_{L-2}^z\rangle\cdots \\ & \cdots\langle S_3^z|e^{-\Delta\tau\mathcal{H}}|S_2^z\rangle\langle S_2^z|e^{-\Delta\tau\mathcal{H}}|S_1^z\rangle, \end{aligned} \tag{4}$$

where l indices the time slice $\tau = l\cdot\Delta\tau$.

Next, we employ the Trotter-Suzuki decomposition,

$$\begin{aligned} \langle S_{l+1}^z|e^{-\Delta\tau\mathcal{H}}|S_l^z\rangle &= \langle S_{l+1}^z|e^{-\Delta\tau\mathcal{H}_1-\Delta\tau\mathcal{H}_0}|S_l^z\rangle \\ &= \langle S_{l+1}^z|e^{-\Delta\tau\mathcal{H}_1}e^{-\Delta\tau\mathcal{H}_0}|S_l^z\rangle + \boldsymbol{O}[(\Delta\tau)^2], \end{aligned} \tag{5}$$

where $\mathcal{H}_0 = J_1\sum_{\langle i,j\rangle}S_i^zS_j^z + J_2\sum_{\langle\langle i,j\rangle\rangle}S_i^zS_j^z$ and $\mathcal{H}_1 = -h\sum_i S_i^x$. Therefore the corresponding matrix element reads

$$\langle S_{l+1}^z|e^{-\Delta\tau\mathcal{H}_1}e^{-\Delta\tau\mathcal{H}_0}|S_l^z\rangle = \Lambda^N e^{-\Delta\tau J_1\sum_{\langle i,j\rangle}S_{i,l}^zS_{j,l}^z-\Delta\tau J_2\sum_{\langle\langle i,j\rangle\rangle}S_{i,l}^zS_{j,l}^z+\gamma\sum_i S_{i,l}^zS_{i,l+1}^z}, \tag{6}$$

with $\gamma = -\frac{1}{2}\ln\tanh(\Delta\tau h)$, and $\Lambda^2 = \sinh(\Delta\tau h)\cosh(\Delta\tau h)$. For a certain configuration $\{S_{i,l}^z\}$, the configurational weight is,

$$\omega\{S_{i,l}^z\} = \left(\prod_l\prod_{\langle i,j\rangle}e^{-\Delta\tau J_1S_{i,l}^zS_{j,l}^z}\right)\left(\prod_l\prod_{\langle\langle i,j\rangle\rangle}e^{-\Delta\tau J_2S_{i,l}^zS_{j,l}^z}\right)\left(\prod_\tau\prod_{\langle l,l'\rangle}\Lambda e^{\gamma S_{i,l}^zS_{i,l'}^z}\right). \tag{7}$$

Now, the 2D quantum problem becomes a (2+1)D classical Ising model, which can be solved by local or global update schemes, both adopted in our practical Monte Carlo samplings.

Within this framework, the physical observables can be evaluated as

$$\langle\hat{O}\rangle \approx \frac{1}{N}\sum_p^N\hat{O}(\{S_i\}_p) \tag{8}$$

where $\{S_i\}_p$ denotes the spin configurations in which the measurement is performed at time p of the Markov chain. Besides, we are also interested in the imaginary time spin-spin correlation function,

$$G(\mathbf{q},\tau)=\frac{1}{N}\sum_{i,j}e^{i\mathbf{q}\cdot\mathbf{r}_{ij}}\langle S_i^z(\tau)S_j^z(0)\rangle, \tag{9}$$

which should be calculated in prior to the spin spectrum $S(\mathbf{q},\omega)$. The latter can be obtained after a stochastic analytical continuation, as detailed in Methods section of the main text.

[1] G. Kresse and J. Furthmüller, "Efficient iterative schemes for ab initio total-energy calculations using a plane-wave basis set," Phys. Rev. B **54**, 11169–11186 (1996).

[2] G. Kresse and D. Joubert, "From ultrasoft pseudopotentials to the projector augmented-wave method," Phys. Rev. B **59**, 1758–1775 (1999).

[3] B. D. Metcalf, "Ground state spin orderings of the triangular ising model with the nearest and next nearest neighbor interaction," Phys. Lett. A **46**, 325–326 (1974).

[4] E. M. Wheeler, R. Coldea, E. Wawrzyńska, T. Sörgel, M. Jansen, M. M. Koza, J. Taylor, P. Adroguer, and N. Shannon, "Spin dynamics of the frustrated easy-axis triangular antiferromagnet $2H$-$AgNiO_2$ explored by inelastic neutron scattering," Phys. Rev. B **79**, 104421 (2009).

[5] Y. Li, S. Bachus, H. Deng, W. Schmidt, H. Thoma, V. Hutanu, Y. Tokiwa, A. A. Tsirlin, and P. Gegenwart, "Partial up-up-down order with the continuously distributed order parameter in the triangular antiferromagnet $\mathrm{TmMgGaO_4}$," Phys. Rev. X **10**, 011007 (2020).

[6] Y. Shen, C. Liu, Y. Qin, S. Shen, Y.-D. Li, R. Bewley, A. Schneidewind, G. Chen, and J. Zhao, "Intertwined dipolar and multipolar order in the triangular-lattice magnet TmMgGaO4," Nat. Commun. **10**, 4530 (2019).
